# The Exploitation of Local *Vitis vinifera* L. Biodiversity as a Valuable Tool to Cope with Climate Change Maintaining Berry Quality

**DOI:** 10.3390/plants10010071

**Published:** 2020-12-31

**Authors:** María Carmen Antolín, María Toledo, Inmaculada Pascual, Juan José Irigoyen, Nieves Goicoechea

**Affiliations:** Grupo de Fisiología del Estrés en Plantas, Departamento de Biología Ambiental, Unidad Asociada al CSIC (EEAD, Zaragoza, ICVV, Logroño), Facultad de Ciencias, Universidad de Navarra, C/Irunlarrea 1, 31008 Pamplona, Spain; mtoledo.5@alumni.unav.es (M.T.); ipascual@unav.es (I.P.); jirigo@unav.es (J.J.I.); niegoi@unav.es (N.G.)

**Keywords:** anthocyanins, elevated CO_2_, high temperature, old grapevine genotypes, phenolic compounds, soluble solids

## Abstract

(1) Background: The associated increase in global mean surface temperature together with raised atmospheric carbon dioxide (CO_2_) concentration is exerting a profound influence on grapevine development (phenology) and grape quality. The exploitation of the local genetic diversity based on the recovery of ancient varieties has been proposed as an interesting option to cope with climate change and maintaining grape quality. Therefore, this research aimed to characterize the potential fruit quality of genotypes from seven local old grapevine varieties grown under climate change conditions. (2) Methods: The study was carried out on fruit-bearing cuttings (one cluster per plant) that were grown in pots in temperature gradient greenhouses (TGG). Two treatments were applied from fruit set to maturity: (1) ambient CO_2_ (400 ppm) and temperature (T) (ACAT) and (2) elevated CO_2_ (700 ppm) and temperature (T + 4 °C) (ECET). (3) Results: Results showed that some of the old genotypes tested remained quite stable during the climate change conditions in terms of fruit quality (mainly, total soluble solids and phenolic content) and of must antioxidant properties. (4) Conclusion: This research underlines the usefulness of exploiting local grapevine diversity to cope with climate change successfully, although further studies under field conditions and with whole plants are needed before extrapolating the results to the vineyard.

## 1. Introduction

Carbon dioxide (CO_2_) is the most important anthropogenic greenhouse gas, which has increased since the pre-industrial period from 280 to 416 µmol mol^−1^ (ppm) in 2019 [1]. Its atmospheric concentration is expected to rise to ca. 700 ppm at the end of this century [2]. Besides this CO_2_ increase, the average air temperature has risen about 0.9 °C since the late nineteenth century, most of that warming took place in the past 35 years [3]. According to the Intergovernmental Panel on Climate Change [4], the increase of global mean temperature by the end of the 21st century (2081–2100), relative to the period (1986–2005), will range from 0.3 °C to 4.8 °C. With these climate projections, the viticulture suitability may be greatly affected in most of the regions of the world [5,6,7], which will need to adapt to changing climatic conditions to continue the production of quality wines at economically sustainable yields [8,9,10].

The increase in global mean temperature is exerting a profound influence on grapevine physiology [10]. For this reason, during the last decade, the consequences of warming on yield and berry quality have been extensively investigated with different approaches. One of the most important consequences linked to the increased ambient temperatures is the shifting of dates and the shortening of the phenological stages [11,12,13]. Moreover, high temperature has a great impact on berry composition, which lead to the production of unbalanced red wines with high alcohol levels, the reduction of acidity, and changes of the phenolic composition of berries [14,15,16,17,18,19,20], all of which, have a noticeable impact on the organoleptic properties that distinguish each variety. On the other hand, most studies focused on the influence of elevated CO_2_ on grapevines show increased vegetative and fruit biomass due to higher rates of photosynthesis, with little repercussions on the quality of grapes and wine [21,22,23,24,25]. However, the effect of elevated CO_2_ stimulating grapevine production were attenuated when elevated CO_2_ was combined with high temperature [21,26]. Within a global change scenario, the ideal grape composition for some specific wines is more difficult to achieve [27].

The wine industry uses a limited number of *Vitis vinifera* L. genotypes, thus in most grape-growing regions, the spread of world-renowned varieties has caused a considerable loss of autochthones grapevine genotypes traditionally grown [28]. Given the future climatic forecasts, it has been proposed the varietal and clonal selection, as well as diversification of wines produced, as valuable tools to regulate a too much accelerated and/or unbalanced grape ripening process [12]. In this view, recent studies have shown that the intra-varietal diversity of commercial varieties could be exploited to maintain grape quality under future climate conditions [18,29,30]. On the other hand, in the last decade, there has been a renewed interest in recovering and studying local ancient cultivars to preserve the genetic resources of each grape-growing region [31,32,33,34]. This trend could be a valuable strategy to sustain higher genetic diversity, as well as product diversity in the markets. To this end, we have recently characterized the oenological potential of some ancient grapevine varieties under greenhouse conditions [35]. To learn more about some of these varieties, under the present study we undertook a characterization of their response to some climate change conditions, in terms of plant growth and fruit composition. Therefore, this work aimed to determine if the biodiversity hidden within local grapevine varieties could be used as a potential tool to help viticulture to adapt to the future climate scenario. The study was performed in potted vines grown in temperature gradient greenhouses (TGGs) facilities, where an elevated CO_2_ treatment of 700 ppm and elevated temperature treatment of +4 °C relative to ambient were imposed, simulating, at least in part, the likely climate conditions around 2100 [2].

## 2. Results

### 2.1. Plant Characteristics

Under our experimental conditions, the genotype was the main factor influencing the length of the phenological stages (Table 1). As expected, Tempranillo (TEMP) showed the shortest ripening period and Tortozona Tinta (TOR) the longest one. However, the length of the ripening was neither affected by climate treatments nor by their interaction (G × T ≥ 0.05).

**Figure 1:**
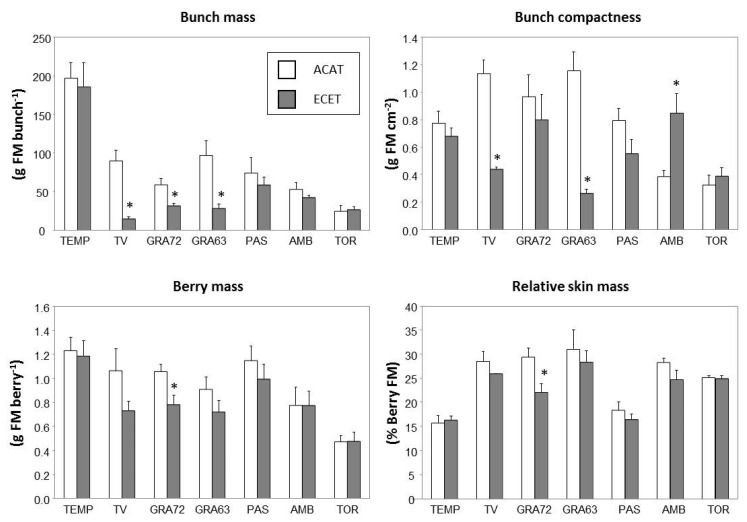
Bunch and berry characteristics from fruit-bearing cuttings of genotypes from seven local old grapevine varieties recovered in Navarre (Spain) grown under two climate scenarios during berry ripening: (1) ambient CO_2_ (400 ppm) and ambient temperature (T) (ACAT) and (2) elevated CO_2_ (700 ppm) and elevated temperature (T + 4 °C) (ECET). Values are means ± S.E. (n = 3–6). Within each genotype, asterisks (*) indicate significant differences (*p* < 0.05) between treatments according to Duncan’s test. Genotype labels can be found in Table 5.

The bunch and berry characteristics significantly differed among genotypes (Table 2). TEMP was the cultivar with the biggest bunch and berry masses, whereas TOR had the lowest bunch and berry sizes. Overall, in plants grown under elevated CO_2_ and high temperature (ECET) bunch mass and compactness, and berry mass were significantly reduced. The effect on bunch compactness depended on the variety, as indicated by the significant interaction observed between factors (G × T, *p* < 0.001). However, the relative skin mass depended mainly on the genotype factor, TEMP and Pasera (PAS) being the ones that achieved the lowest values. Tinto Velasco (TV), Graciano 72 (GRA72), and Graciano 63 (GRA63) were the more responsive genotypes to ECET treatment, in terms of bunch size (Figure 1). TV and GRA63 showed significant reductions in bunch compactness in ECET compared with ACAT, whereas for AMB, bunch compactness was higher in the ECET treatment. In GRA72, bunch and berry mass, and relative skin mass under ECET conditions were lower than under ACAT.

### 2.2. Berry Composition

**Figure 2:**
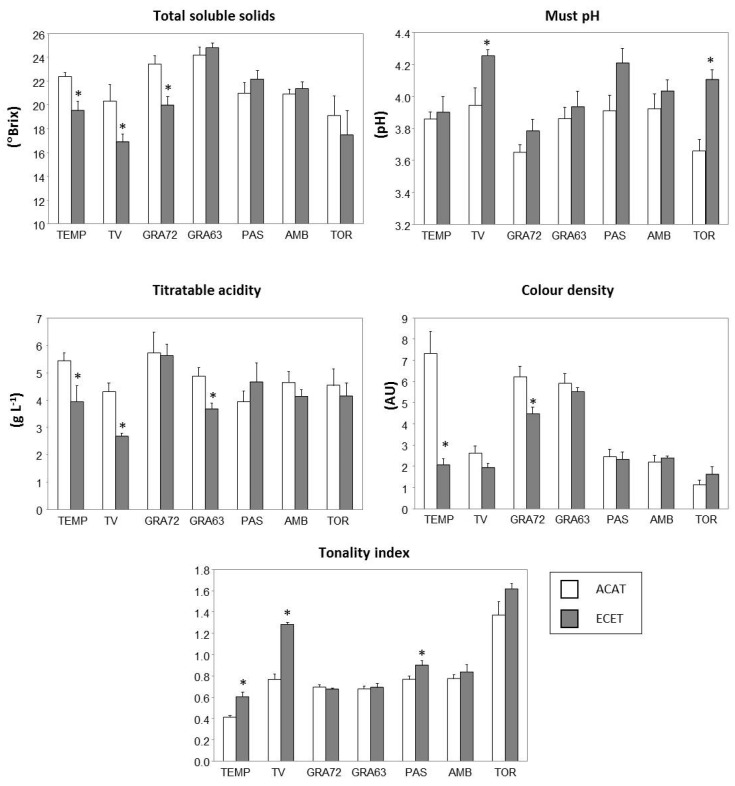
Must characteristics from fruit-bearing cuttings of genotypes from seven local old grapevine varieties recovered in Navarre (Spain) grown under two climate scenarios during berry ripening: (1) ambient CO_2_ (400 ppm) and ambient temperature (T) (ACAT) and (2) elevated CO_2_ (700 ppm) and elevated temperature (T + 4 °C) (ECET). Values are means ± S.E. (n = 3–6). Within each genotype, asterisks (*) indicate significant differences (*p* < 0.05) between treatments according to Duncan’s test. Genotype labels can be found in Table 5.

The genotype was the main factor in modifying all must characteristics (Table 3). Overall, the ECET treatment resulted in a low concentration of total soluble solids and titratable acidity and in a high must pH. The effect on total soluble solids had different intensities depending on the genotype, as indicated by the significant interaction between factors (G × T, *p* < 0.05). Under ECET conditions, the accumulation of sugars was significantly decreased in TEMP, TV, and GRA72, and no changes were detected in GRA63, PAS, Ambrosina (AMB), and TOR (Figure 2). The ECET condition increased must pH and reduced titratable acidity in TV, whereas those respective changes on both parameters were not clearly associated in the case of TOR, TEMP, and GRA63.

Altogether, the color density of must was significantly decreased under the ECET scenario, whereas the tonality index increased (Table 3). However, the observed changes depended on the genotype, as indicates the significant interaction between these factors (G × T, *p* < 0.001). Indeed, color density was strongly reduced in TEMP and to a lesser extent in GRA72, whereas the tonality index increased in TEMP, TV, and PAS (Figure 2). The chromatic characteristics of must of GRA63, AMB, and TOR were not modified by the ECET treatment.

**Figure 3:**
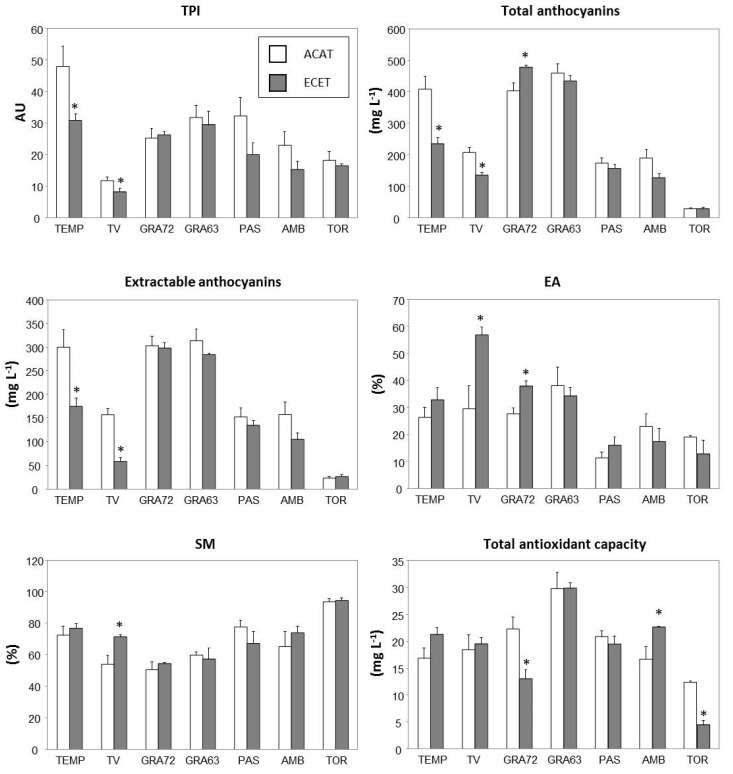
Phenolic composition and antioxidant capacity from fruit-bearing cuttings of genotypes from seven local old grapevine varieties recovered in Navarre (Spain) grown under two climate scenarios during berry ripening: (1) ambient CO_2_ (400 ppm) and ambient temperature (T) (ACAT) and (2) elevated CO_2_ (700 ppm) and elevated temperature (T + 4 °C) (ECET). Values are means ± S.E. (n = 3–6). Within each genotype, asterisks (*) indicate significant differences (*p* < 0.05) between treatments according to Duncan’s test. Genotype labels can be found in Table 5.

Regarding phenolic composition, TEMP had the highest values of total phenolic content (TPI), whereas TV and TOR had the lowest TPI in berries (Table 4). In general, TPI decreased in plants subjected to ECET conditions, these changes being significant only in TEMP and TV (Figure 3). GRA72 and GRA63 were the genotypes with the highest concentration of anthocyanins whereas, as expected, TOR was the variety with lower anthocyanin content (pinked genotype) (Table 4). The total anthocyanin content was also significantly modified under the ECET scenario, the extent of the effect being dependent on the variety (G × T, *p* < 0.001) (Table 4). Under ECET conditions, the total anthocyanin content significantly decreased in TEMP and TV whereas GRA72 was the sole genotype in which anthocyanin accumulation was improved under climate change conditions (Figure 3). Similarly, a clear interaction between the two factors was observed for extractable anthocyanins and their extractability (EA) (G × T, *p* < 0.01). Although there was not a consistent effect of ECET treatment over EA in all the genotypes assessed, significant increases of this parameter were detected in TV and GRA72 (Figure 3). ECET conditions did not affect the seed maturity (SM) index except for the TV, in which SM significantly increased. Finally, the DPPH assay performed in must to test its total antioxidant capacity showed that GRA63 was the genotype with the highest antioxidant capacity, TOR being the variety with the lowest values (Table 4). Besides, a significant interaction between genotype and treatment was observed for this parameter (G × T, *p* < 0.001). Consequently, total antioxidant capacity significantly decreased in GRA72 and TOR while it significantly increased in AMB, under ECET conditions (Figure 3).

Considering the two main factors studied, variety and climate change, the variety was the factor with a higher influence on the grape composition. A clear distinction was observed between GRA72, GRA63, and TEMP with respect to TV, AMB, and TOR, as can be observed in the PCA (Figure S1A). Anthocyanin content (total and extractable), tonality index, color density, and TPI, to a larger extent, but also of the phenological cycle, the total soluble solids and, bunch and berry mass to a lesser extent, explained variance across varieties (Figure 1, Figure 2 and Figure 3 and Figure S1B). Regarding the impact of environmental conditions, TEMP and TV seemed to be the varieties more affected, as indicates the higher separation between ACAT and ECET points in both cases (Figure S1A). This separation was mainly associated with changes in anthocyanin levels (Table 4) and color properties of the must (Table 3), as well as to alterations in the phenology of the bunch (Table 1).

## 3. Discussion

The adaption capacity of ancient grapevine varieties to climatic conditions has been suggested as a valuable tool to exploit the grapevine diversity in relation to traits that are impacted by climate change, such as phenology, the accumulation of sugars, organic acids, and phenolic compounds [35,36,37]. The hypotheses raised in this study were: (i) the genotypes studied performed differently according to the parameters analyzed; (ii) the distinctive properties of each genotype can evolve differently in the new climate scenario. The seven genotypes from old varieties tested are fully distinguished by features as the length of the reproductive cycle, bunch and berry mass, total soluble solids, TPI, and anthocyanin content (Figure S1). According to our results, TEMP, GRA72, and GRA63 stood out for having higher anthocyanin content, color density, and TPI, whereas TV, AMB, PAS, and TOR were characterized by presenting longer veraison to maturity and fruit set to maturity periods.

A growing body of evidence shows that grapevine phenology is hastened by elevated temperatures [12,13,38] and high atmospheric CO_2_ concentration [13,39]. Warmer temperature joint to elevated CO_2_ concentrations also produced, in general, a faster grapevine development but the effect had different intensity depending on the commercial clone tested [39,40,41]. By contrast, in our study, the ECET treatment did not modify the length of phenological phases in the genotypes from old varieties tested being. TEMP was the earlier ripening variety and TOR the latest ripening regardless of the treatment applied (Table 1). A long-term study from Biasi et al. [36] shows that climate change modified the phenology of autochthonous grapevine varieties to a lesser extent than that of international varieties, which agrees partially with our results. However, it cannot discard that the artificial conditions of the TGGs and the relatively short duration of elevated CO_2_ treatment of our study had also influenced the results. In this last aspect, Edwards et al. [23] indicated that whereas elevated temperature impacted phenology from the onset of treatment, the effects of elevated CO_2_ only started to be detected after three years of treatment.

Most studies have reported that the effects of elevated CO_2_ on grapevines are more related to bunch and berry mass than to fruit quality. In general, increasing CO_2_ led to increasing of the bunch and berry mass [21,22,24,25] and this effect was maintained when elevated CO_2_ interacted with high temperature [26]. Our results are in contrast with those of Kizildeniz et al. [26] since that ECET treatment resulted in a general reduction of bunch mass and compactness, and berry mass (Table 2). Those discrepancies could be explained, at least partially, by differences in the ambient temperatures of each growing season (five heatwaves recorded in the ECET treatment), which could have become the main factor contributing to the reduction of yield detected in our study [29,40]. Regarding the genotypes, TV, GRA72 and GRA63 were the most affected by ECET treatment in terms of yield, whereas no changes were detected in bunch or berry characteristics of TEMP, PAS, AMB, and TOR. These results confirm a broad range of responses to changes in environmental conditions among the genotypes from old varieties assessed (Figure 1). In addition, ECET treatment only reduced the relative skin mass in GRA72. This change could have relevance because the structure of berry skin plays a key role as a constitutive defense barrier against pathogens (for example, fungi) that try to invade grapes. It has been found that warm conditions favoring the development of fungi can also reduce the reinforcement of the berry skin, thus increasing the susceptibility of fruits to be penetrated by fungi growing on their surface [42].

The rise of air temperature affects gene expression and enzymatic activity of primary and secondary metabolism of grape berries, which could have a considerable impact on must and wine characteristics [19]. The main effects of warming on berry composition include reduction of anthocyanin content [14,20,29], fall of organic acids [15], and changes in the composition of phenolic compounds, mainly anthocyanins and flavonols [18,43]. In addition, high temperatures hasten the accumulation of sugars in the must, leading to the elaboration of wines with higher alcohol content [11]. However, the extent of all described impacts on berry quality differs among varieties [14,20] and even, among clones within the same variety [18,29,30]. Until now, all these studies have focused on commercial varieties, so studying the behavior of old varieties can provide valuable information to assess their use in a future climate scenario. In fact, our results suggest that, under ECET conditions, the old grapevine genotypes could respond quite distinctly than the reported commercial ones. So, under ECET, the accumulation of sugars was significantly decreased in some cases (TEMP, TV, and GRA72), and no changes were detected in the others (GRA63, PAS, AMB, and TOR) (Figure 2). These results contrast with general observations that sugar accumulation is largely increased by warm temperatures, such as occurred in commercial clones of Tempranillo [29], Chardonnay, Shiraz, and Cabernet Sauvignon [44]. In addition, it has been demonstrated that increasing CO_2_ has little effect on total soluble solids in commercial varieties like Riesling and Cabernet Sauvignon [25] and Tempranillo [26]. However, in our case, it is possible that the effect of elevated temperature was compensated when the temperature interacted with elevated CO_2_. Moreover, it should be taken into account that in the present study, the harvest dates were based on both the total soluble solids and titratable acidity, leading to an early harvest of some genotypes to avoid the excessive loss of acidity (see Material and Methods section). Although comparisons of the behavior of potted plants grown under our artificial conditions with field grapevines are not direct, our results suggest that, under the future climatic scenario, some of the old genotypes tested would be able to control better the sugar accumulation rates than some commercial genotypes grown in similar artificial conditions [26,29] (Figure 2).

Like sugars, titratable acidity reflects the degree of berry ripening. In grapes, the acidity depends on levels of both tartaric and malic acid, whose pathways can be connected [45]. The general trend is that high temperatures accelerate the decrease of grape acidity during ripening, mainly because of the faster depletion of malic acid leading to potential effects on wine aging capacity [46]. Thereby, it was reported that high temperatures produced malate losses in commercial varieties as Shiraz [15], Cabernet Sauvignon, Chardonnay [14], and Tempranillo [29]. In addition, studies in which the elevated CO_2_ was combined with high temperature have reported decreases of malic acid in different commercial clones of Tempranillo [39,41]. Our results partially agree with those described in commercial varieties because some old genotypes (TEMP, TV, and GRA63) also experienced significant decreases of titratable acidity in response to ECET treatment (Figure 2).

Some research has indicated that, under a global warming scenario, the decoupling between the accumulation of primary metabolites (namely, sugars and organic acids) and the secondary metabolites (i.e., phenolic and aromatic compounds) in berries will be accentuated [16,30]. Among these secondary metabolites, berry phenolic compounds contribute to the organoleptic properties of the wine (i.e., taste, color, and aroma) and, in addition, they have benefits to human health due to their antioxidant capacity [47,48]. The phenolic compounds most reduced under elevated temperatures are anthocyanins and flavonols [18,41,49], which lead to a decrease in the total phenolic content of berries. In our experimental conditions, the genotypes of old grapevine varieties displayed different trends since the ECET treatment caused the decrease of TPI and total anthocyanins in TEMP and TV, the maintenance of these compounds in GRA63, PAS, AMB, and TOR, and the improvement of anthocyanin content in GRA72 (Figure 3). The reduction of phenolic content has been related to decreases in enzymatic activities involved in the phenylpropanoid pathway [20]. Other studies confirmed the degradation of anthocyanins by peroxidases, which exhibit higher activity at elevated temperatures [49,50,51]. In addition, the increase in the tonality index of TEMP, TV, and PAS points to significant modifications of must characteristics from these varieties that could be associated with differences in anthocyanin profiles (Figure 2). Alterations in the relative abundance of different anthocyanin families are known to lead to different tonalities [52]. Grape phenolics have a variable extraction potential (called extractability), whose assessment is based on anthocyanin extraction from the whole berries [53]. Our research includes the measurement of extractable anthocyanins and estimations of cellular extractability of anthocyanins (EA) and seed maturity (SM) to estimate the impact of climate change on the oenological potential of each variety. Overall, EA and SM were little affected by the combination of elevated CO_2_ and high temperature (Table 4). However, under ECET treatment, EA increased in GRA72 and, especially in TV, indicating that the lower potential of color extraction of this genotype could get worse under ECET conditions (Figure 3) [35]. The SM was a stable property that only increased in TV under ECET conditions but staying within the average values of the assessed genotypes. High values of SM are typical of seeds with non-polymerized tannins, which give high green astringency to the resulting wine [53]. This was the case of the TOR whose SM values were excessively high, regardless of the temperature and CO_2_ condition (Table 4).

Phenolic compounds have received considerable interest based on their antioxidant and free-radical-scavenging properties, catechins, proanthocyanidins, and anthocyanins being the most abundant antioxidants present in berries [48,54]. Our data show that, apart from GRA63, the total antioxidant capacity of old genotypes was lower than that of commercial clones of Tempranillo [29]. So, GRA63 was the genotype with the highest antioxidant capacity whereas TOR was the genotype with the lowest values, which could be explained, at least in part, by the considerable difference in the anthocyanin content between both genotypes (Table 4). A significant relationship between antioxidant capacity and anthocyanin content has been reported in different grapevine varieties [55,56,57]. By contrast, under the ECET treatment imposed in our study, neither the decrease in total antioxidant capacity of GRA72 and TOR nor the improvement of this property in AMB did seem to be directly related to changes in TPI and/or anthocyanins (Figure 3), suggesting that the antioxidant potential could be more related to specific phenolic compounds rather than to the total concentrations [57].

Finally, it should be taken into account that the old genotypes assessed in the present study showed the different length of reproductive phases, which could have conditioned some responses to the combination of elevated CO_2_ and high temperature in terms of berry quality (Table 1). Thus, the harvest took out under quite different temperatures regardless of the CO_2_ treatment applied, ranging from 36 °C in the middle of August (harvest of TEMP) to 29 °C at the end of October (harvest of TOR) (Figure 5). In addition, we have recorded that under ECET, 57% of the ripening period of TEMP occurred at temperatures above 35 °C, which could have accentuated, at least in part, the susceptibility of this genotype to imposed conditions.

## 4. Materials and Methods

### 4.1. Biological Material and Growth Conditions

The local grapevine genotypes included in this study are a selection of more than 65 genotypes recovered, from 2002 to 2020, in old vineyards (older than 65 years), and identified using molecular markers [58]. These genotypes were multiplied and conserved in the germplasm bank of the Estación de Viticultura y Enología de Navarra (EVENA). The selection was based on the agronomic characterization performed by EVENA (unpublished data). Genotypes with oenological potential and that differed in their phenological cycle, bunch mass, and berry mass were chosen for the study (Table 5). This set of varieties consisted of seven old grapevine genotypes (*Vitis vinifera* L.) growing in an experimental vineyard located in Olite (Navarra, Spain) (latitude: 42°29′15″ N; longitude: 1°39′45″ W; altitude: 388 mamsl). Fifty dormant cuttings of each genotype were collected after the winter pruning of 2018. The 400–500 mm long cuttings were induced for fruit-bearing according to the steps originally outlined by Mullins [59] and Ollat et al. [60]. Rooting was induced by immersing the cuttings in a solution of indole-3-butyric acid (400 mg L^−1^) and placing them in a warm bed (27 °C) in a cold room (4 °C) for 30 days. Once rooting was successful, the cuttings were planted in 0.8 L plastic pots containing perlite and peat (1:1 v:v) and then, were transferred to a greenhouse. Initial growth conditions were 25/15 °C and 50/90% relative humidity (day/night) regime and natural daylight (photosynthetic photon flux density, PPFD, was on average 850 μmol m^−2^ s^−1^ at midday) supplemented with high-pressure sodium lamps (SON-T Agro Phillips, Eindhoven, The Netherlands) to extend the photoperiod up to 15 h. Under these conditions, bud-break took place after 7–8 days (day of the year (DOY) 105) and from this moment the growth was controlled until flowering (DOY 151) leaving only one inflorescence and 4 leaves per plant.

### 4.2. Experimental Design

**Figure 4:**
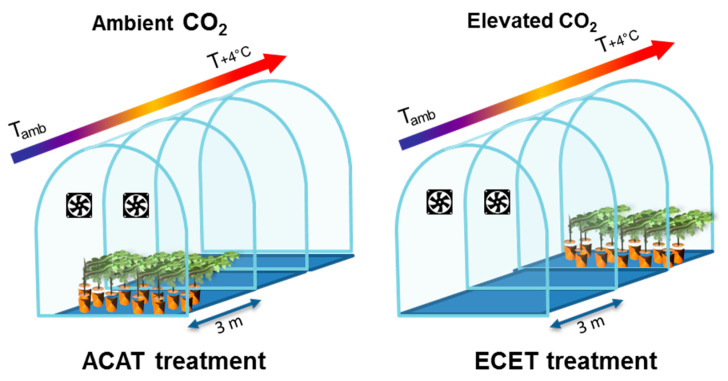
The modular design of TGGs. A gradient of temperature is created from module 1 of ambient temperature to module 3 of ambient temperature +4 °C. In ACAT treatment, plants were placed in module 1 of the TGG with ambient CO_2_. In ECET treatment, plants were placed in module 3 of the TGG with elevated CO_2_.

After fruit set (Eichhorn and Lorenz (E-L) growth stage 27) [61] that took place about 50 days after bud-break, plants were transplanted to 13 L plastic pots containing perlite and peat (1:1 v:v). From this moment, plants grew freely until reaching 14 leaves per plant. Afterward, the vegetative growth was controlled by pruning to maintain a leaf area to fruit mass ratio adequate for berry ripening [62]. Afterward, plants were transferred to four temperature gradient greenhouses (TGG) located at the University of Navarra (Pamplona, Spain) (latitude: 42°49′00″ N; longitude: 1°39′00″ W; altitude: 450 mamsl) for the application of the CO_2_ and temperature treatments. TGGs were built with a modular design with three temperature modules (3.04 m long each) (Figure 4). Within each TGG a temperature gradient is created (from module 1 of ambient temperature to module 3 of ambient temperature +4 °C) by circulating air to maintain the difference of 4 °C between modules (more details in Morales et al. [63]). Module 2 had no plants because it is a module of transition. To increase the concentration of CO_2_, the gas is injected inside the modules until the desired concentration is attained. Inside the TGGs, pots were placed in holes made in the soil to ensure natural temperature fluctuations at the root zone.

Plants of all varieties were randomly distributed into the TGGs, and we established two climate conditions in two TGGs for each one: (1) ambient CO_2_ (ca. 400 µmol mol^−1^) and ambient temperature (T) (ACAT) and (2) elevated CO_2_ (ca. 700 µmol mol^−1^) and elevated temperature (T + 4 °C) (ECET). Plants of treatment ACAT were placed in module 1 whereas plants of treatment ECET were placed in module 3 of the TGG with the desired concentration of CO_2_ (Figure 4). There were 3–6 replicates depending on genotype making a total of 34 plants for each treatment (ACAT and ECET) The irrigation (both at the pre-treatment greenhouse and at the TGGs) was performed using the nutritive solution described by Ollat et al. [60] alternated with deionized water. Plants were watered twice per day and irrigation doses ranged between 2.5 and 3.5 L. according to the needs of the plant during fruit ripening. Quartz stones were used on the surface of the pots to avoid evaporation and with it, excessive loss of water from the substrate. Plants remained in the TGGs until the berries reached commercial maturity (E-L 38 stage).

### 4.3. Weather Conditions

**Figure 5:**
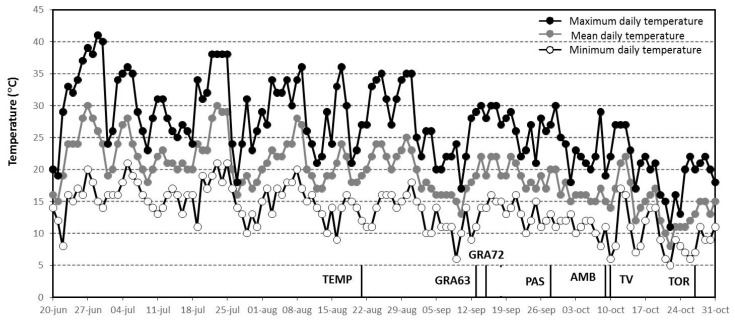
Time course of minimum, maximum, and average air temperature recorded from fruit set to maturity of berries. Bars indicate the average harvest date of each genotype. Genotype labels can be found in Table 5.

The minimum, mean, and maximum daily temperatures from 20 June (DOY 171) to 31 October (DOY 304) were recorded during the growing season covered by the experiment. Weather data were recorded from the Pamplona Airport station (Navarra, Spain) and the reference period 1999 to 2019 (AEMET, Spain). The growing season of 2019 was warmer compared to the reference data for the same period within the last 20 years (Table 6). Thereby, the maximum daily air temperature was between 2 and 6 °C higher than the average registered within the 2009–2019 period. Particularly, June was extremely hot (peak temperature of 41.0 °C) (Figure 5) whereas the minimum daily air temperature of October was 3 °C higher than the average registered for the same month during the period 2009–2019. In addition, the number of extreme temperature events differed between the two temperature treatments, 15 and 26 days with maximum temperature above 35 °C in the ambient temperature treatment (ACAT) (Figure 5) and in the elevated temperature treatment (ECET) (data are not shown because followed the same trends plus 4 °C), respectively. Hayman et al. [64] establish as a heatwave either five consecutive days with maximum daily air temperatures above 35 °C or three consecutive days with maximum daily air temperatures above 40 °C. According to this definition, in the ECET treatment, five and one heatwaves were recorded during the ECET and ACAT treatments, respectively.

### 4.4. Phenology and Berry Determinations

The length of phenological phases was recorded independently for each plant as the number of days from fruit set (E-L 27 stage) to when 10% of the berries of the bunch were colored (E-L 35 stage, veraison), and from veraison (E-L 35 stage) to maturity (E-L38 stage). Fruit set and veraison dates were assessed visually. Following the expert’s advice from the EVENA, every plant was harvested when the ratio of sugars to acidity ranged between 4 and 6.

At harvest, the weight and length of each bunch were measured. Both data were used to calculate the bunch compactness, which was expressed as bunch mass-to-squared length ratio [65]. Ten berries from each plant were collected and weighed to obtain the mean fresh berry mass (in grams). Then, the same berries were separated into skin and flesh. The relative skin mass was calculated as the quotient between skin fresh matter (FM) and total berry FM expressed as a percentage. The remaining berries of each plant were frozen at −20 °C (around two months) for further analysis.

### 4.5. Berry Quality Determinations

At maturity, a subsample of 20 berries of each plant was triturated and then was centrifuged at 4100× *g* at 4 °C for 10 min. The supernatant was used for the following determinations: total soluble solids measured with a temperature-compensating refractometer (Zuzi model 315; Auxilab, Beriáin, Spain) and expressed as °Brix; must pH measured with a pH meter (Crison Instruments, Barcelona, Spain) standardized to pH 7.0 and 4.0; titratable acidity measured by titration with NaOH according to International Organization of Vine [66] and expressed as g tartaric acid per L of the must. To analyze the content of anthocyanins, total phenols, and chromatic properties, another 20-berry subsample of each plant was taken. Total and extractable anthocyanins were following the procedure of Saint-Cricq et al. [67]. Two aliquots of the non-filtered, crushed grape homogenate were macerated for 4h at pH 1 (hydrogen chloride) and pH 3.2 (tartaric acid), respectively. Then, the macerated samples were centrifuged at 4100× *g* at 4 °C for 10 min. Total and extractable anthocyanins were determined in both supernatants (macerated at pH 1 and pH 3.2) by reading absorbance at 520 nm and the results were expressed as mg per mL [68]. Both data were used to calculate the cellular extractability of anthocyanins (EA) [53]. Total polyphenol index (TPI) was determined by the absorbance reading at 280 nm in the supernatant obtained after maceration at pH 3.2 [69]. The seed maturity (SM) index was calculated from TPI and extractable anthocyanins values [53]. Color density was obtained from the sum of the absorbance readings at 420, 520, and 620 nm, whereas tonality index was calculated as the ratio of absorbance readings at 420 and 520 nm of the samples extracted at pH 3.2 [70].

### 4.6. Total Antioxidant Capacity

Total antioxidant capacity was evaluated on the same must samples used for berry quality determinations by using the free-radical scavenging activity (α,α-diphenyl-β-picrylhydrazyl, DPPH) assay [71]. The reaction was started by adding 25 µL of the sample to the cuvette containing 80 µM (methanol solution) (975 µL) of the free radical (DPPH•). Samples were incubated at 25 °C for 15 min, after which the absorbance at 515 nm was read. The calibration curve was made using gallic acid as a standard and results were expressed as mg gallic acid per mL of the must.

### 4.7. Statistical Analyses

Statistical analyses were carried out using the Statistical Package for the Social Sciences (SPSS) software (SPSS Inc., Chicago, IL, USA) version 22.0 for Windows. The principal component analysis (PCA) was conducted to determine general trends of different genotypes and treatments. This analysis provided a tool to describe the main differences among the genotypes studied in terms of plant characteristics and berry composition, as well as to identify the variables involved in their response to climate change. Bartlett’s test of sphericity and the Kaiser–Meyer–Olkin (KMO) test were calculated to assess the suitability of the data to PCA. Then, an analysis of variance (ANOVA) was employed once proved that the data met the assumptions of normality (Shapiro–Wilks test) and homoscedasticity (Levene’s test) with a threshold of 0.05. When ANOVA was statistically significant (*p* < 0.05), the differences among groups were tested with a Duncan test post-hoc test. Results were considered statistically significant if *p* < 0.05.

## 5. Conclusions

The old varieties studied showed a differential response to the future scenario of climate change defined as a combination of elevated atmospheric CO_2_ and high temperature. The experiment was done with grapevine fruit-bearing cuttings (one cluster per plant), grown in pots and under greenhouse conditions. Under these artificial experimental conditions, TEMP and TV were the varieties that showed lower stability, especially concerning berry traits such as anthocyanin content and must color, thus showing a higher degree of phenotypic plasticity in response to changes in air temperature and CO_2_. Such higher plasticity may limit the use of these cultivars under future climate scenarios. However, genotypes such as GRA63, PAS, and AMB remained quite stable during the climate change conditions in terms of fruit quality (mainly, total soluble solids and phenolic content) and of antioxidant properties, which all is indicative that these genotypes could be exploited to cope with some constraints related to climate change as increasing air temperature and CO_2_. This research reveals the importance of testing the performance of the local old grapevine varieties under future climate conditions adding new knowledge to exploit such biodiversity. The present study also offers the first data on the adaptive potential to climate change of old varieties of grapevines. However, given the limitations of the experiment (potted plants with only one cluster, small berry sampling size, and greenhouse conditions) further studies under natural conditions (whole plants, Free Air Carbon Dioxide Enrichment (FACE) in the field) are required before extrapolating the present results to vineyards.

## Figures and Tables

**Table 1:** Phenology from fruit-bearing cuttings of genotypes from seven local old grapevine varieties recovered in Navarre (Spain) grown under two climate scenarios during berry ripening: (1) ambient CO_2_ (400 ppm) and ambient temperature (T) (ACAT) and (2) elevated CO_2_ (700 ppm) and elevated temperature (T + 4 °C) (ECET). Genotype labels can be found in Table 5.

Varieties	Fruit Set-Veraison (Days)	Veraison-Maturity (Days)	Fruit Set-Maturity (Days)
TEMP	52 d ^1^	35 d	87 e
TV	70 b	67 bc	137 b
GRA72	68 b	42 d	110 d
GRA63	67 bc	46 d	113 cd
PAS	66 bc	58 c	124 c
AMB	61 c	80 a	141 b
TOR	86 a	71 ab	157 a
Treatments			
ACAT	65 a	56 a	121 a
ECET	66 a	59 a	125 a
ANOVA ^2^			
Genotype (G)	***	***	***
Treatment (T)	ns	ns	ns
G × T	ns	ns	ns

^1^ Values represent means. Within columns, means followed by the same letter do not differ significantly (*p* ≥ 0.05) according to Duncan’s test as affected by the main factors genotype (G) (n = 8–12), treatment (T) (n = 34) and their interaction (G × T). ^2^ Significance of the analysis of variance (ANOVA): *** *p* < 0.001; ns, not significant (*p* ≥ 0.05).

**Table 2:** Bunch and berry characteristics from fruit-bearing cuttings of genotypes from seven local old grapevine varieties recovered in Navarre (Spain) grown under two climate scenarios during berry ripening: (1) ambient CO_2_ (400 ppm) and ambient temperature (T) (ACAT) and (2) elevated CO_2_ (700 ppm) and elevated temperature (T + 4 °C) (ECET). Genotype labels can be found in Table 5.

Varieties	Bunch Mass (g FM Bunch^−1^)	Bunch Compactness (g FM cm^−2^)	Berry Mass (g FM Berry^−1^)	Relative Skin Mass (% Berry FM)
TEMP	191.1 a ^1^	0.73 a	1.21 a	16.0 c
TV	52.2 bc	0.79 a	0.90 bc	27.2 ab
GRA72	45.4 bc	0.88 a	0.92 bc	25.8 b
GRA63	62.5 bc	0.71 a	0.81 bc	29.7 a
PAS	66.4 b	0.67 a	1.07 ab	17.4 c
AMB	47.8 bc	0.62 ab	0.77 c	26.5 ab
TOR	25.8 c	0.36 b	0.47 d	25.1 b
Treatments				
ACAT	89.4 a	0.78 a	0.97 a	24.7 a
ECET	61.2 b	0.59 b	0.83 b	22.3 a
ANOVA ^2^				
Genotype (G)	***	***	***	***
Treatment (T)	***	***	*	ns
G × T	ns	***	ns	ns

^1^ Values represent means. Within columns, means followed by the same letter do not differ significantly (*p* ≥ 0.05) according to Duncan’s test as affected by the main factors genotype (G) (n = 8–12), treatment (T) (n = 34) and their interaction (G × T). ^2^ Significance of the analysis of variance (ANOVA): * *p* < 0.05; *** *p* < 0.001; ns, not significant (*p* ≥ 0.05). FM indicates fresh matter.

**Table 3:** Must characteristics from fruit-bearing cuttings of genotypes from seven local old grapevine varieties recovered in Navarre (Spain) grown under two climate scenarios during berry ripening: (1) ambient CO_2_ (400 ppm) and ambient temperature (T) (ACAT) and (2) elevated CO_2_ (700 ppm) and elevated temperature (T + 4 °C) (ECET). Genotype labels can be found in Table 5.

Varieties	Total Soluble Solids (°Brix)	Must pH	Titratable Acidity (g L^−^^1^)	Color Density (AU)	Tonality Index
TEMP	21.0 b ^1^	3.88 bc	4.69 b	4.70 a	0.51 d
TV	18.6 c	4.10 a	3.49 c	2.29 b	1.03 b
GRA72	21.7 b	3.72 c	5.68 a	5.36 a	0.69 c
GRA63	24.5 a	3.90 abc	4.28 bc	5.72 a	0.69 c
PAS	21.6 b	4.06 ab	4.31 bc	2.40 b	0.84 c
AMB	21.1 b	3.98 ab	4.40 bc	2.31 b	0.81 c
TOR	18.3 c	3.88 bc	4.35 bc	1.39 b	1.49 a
Treatment					
ACAT	21.6 a	3.84 b	4.77 a	4.00 a	0.76 b
ECET	20.3 b	4.04 a	4.10 b	2.79 b	0.93 a
ANOVA ^2^					
Genotype (G)	***	***	**	***	***
Treatment (T)	**	***	*	***	***
G × T	*	ns	ns	***	***

^1^ Values represent means. Within columns, means followed by the same letter do not differ significantly (*p* ≥ 0.05) according to Duncan’s test as affected by the main factors genotype (G) (n = 8–12), treatment (T) (n = 34) and their interaction (G × T). ^2^ Significance of the analysis of variance (ANOVA): * *p* < 0.05; ** *p* < 0.01; *** *p* < 0.001; ns, not significant (*p* ≥ 0.05). AU indicates absorbance units.

**Table 4:** Phenolic composition and antioxidant capacity from fruit-bearing cuttings of genotypes from seven local old grapevine varieties recovered in Navarre (Spain) grown under two climate scenarios during berry ripening: (1) ambient CO_2_ (400 ppm) and ambient temperature (T) (ACAT) and (2) elevated CO_2_ (700 ppm) and elevated temperature (T + 4 °C) (ECET). Genotype labels can be found in Table 5.

Varieties	TPI (AU)	Total Anthocyanins (mg L^−^^1^)	Extractable Anthocyanins (mg L^−^^1^)	EA (%)	SM (%)	Total Antioxidant Capacity (mg L^−^^1^)
TEMP	39.3 a ^1^	321.8 b	237.1 b	29.5 bc	74.6 b	19.0 b
TV	10.0 d	171.6 c	107.9 c	43.1 a	62.7 bcd	18.9 b
GRA72	25.7 bc	440.6 a	300.2 a	32.7 ab	52.5 cd	17.6 b
GRA63	30.6 b	446.8 a	298.5 a	36.1 ab	58.6 d	29.8 a
PAS	26.1 bc	165.4 c	143.4 c	13.7 d	72.4 b	20.2 b
AMB	19.1 c	158.5 c	131.0 c	20.1 cd	69.5 bc	19.7 b
TOR	17.3 cd	29.0 d	24.9 d	15.9 d	90.8 a	8.4 c
Treatment						
ACAT	27.8 a	266.5 a	211.5 a	24.6 a	67.6 a	18.8 a
ECET	20.7 b	217.7 b	149.1 b	29.5 a	71.2 a	18.3 a
ANOVA ^2^						
Genotype (G)	***	***	***	***	***	***
Treatment (T)	**	***	***	ns	ns	ns
G × T	ns	***	**	**	ns	***

^1^ Values represent means. Within columns, means followed by the same letter do not differ significantly (*p* ≥ 0.05) according to Duncan’s test as affected by the main factors genotype (G) (n = 8–12), treatment (T) (n = 34) and their interaction (G × T). ^2^ Significance of the analysis of variance (ANOVA): ** *p* < 0.01; *** *p* < 0.001; ns, not significant (*p* ≥ 0.05). TPI, total polyphenol index; EA, cellular extractability of anthocyanins; SM, seed maturity; AU, absorbance units.

**Table 5:** Summary of the characteristics of the genotypes from seven local old grapevine varieties used in this study. Data provided by the Estación de Viticultura y Enología de Navarra (EVENA) (Navarra, Spain) were collected in 2019 from plants grown in the vineyard.

Genotype	Clone	Code	Reproductive Cycle	Color	Bunch Mass (g Bunch^−1^)	Berry Mass (g)
Tempranillo	T24	TEMP	Short	Red	145	1.71
Tinto Velasco	T73	TV	Medium	Red	153	2.01
Graciano	T72	GRA72	Medium	Red	81	1.13
Graciano	T63	GRA63	Long	Red	74	1.06
Pasera	T85	PAS	Long	Red	331	2.02
Ambrosina	T46	AMB	Long	Red	205	1.38
Tortozona Tinta	T20	TOR	Long	Pink	241	1.21

**Table 6:** Temperature conditions during the growing season of 2019 and the average for the same period in the last 20 years (1999–2019).

Month	June	July	August	September	October
Year	Mean daily air temperature (°C)				
2019	19.0	22.0	22.0	18.0	15.0
1999–2019	19.8	21.5	21.8	18.7	14.9
	Minimum daily air temperature (°C)				
2019	4.0	10.0	9.0	6.0	5.0
1999–2019	7.6	10.0	10.2	6.0	1.9
	Maximum daily air temperature (°C)				
2019	41.0	38.0	36.0	30.0	29.0
1999–2019	35.4	36.8	37.0	32.5	27.3
	Days with temperatures over 35 °C				
2019	5	5	2	0	0

Data recorded from the Pamplona Airport station (Navarra, Spain) were provided by AEMET (Spain).

## Data Availability

Data files of the present study have been deposited in the Department of Environmental Biology (University of Navarra).

## Supplementary Materials

The following are available online at https://www.mdpi.com/2223-7747/10/1/71/s1, Figure S1: Principal component analysis score and loading plot obtained from the statistical analysis of plant and berry characteristics.
