# Supplementary material for: The Exploitation of Local Vitis vinifera L. Biodiversity as a Valuable Tool to Cope with Climate Change Maintaining Berry Quality
Source: Plants (Basel). 2020 Dec 31;10(1):71. doi: 10.3390/plants10010071 (PMC7824074; doi:10.3390/plants10010071)
Supplement: Supplementary file 1 [file plants-10-00071-s001.pdf]

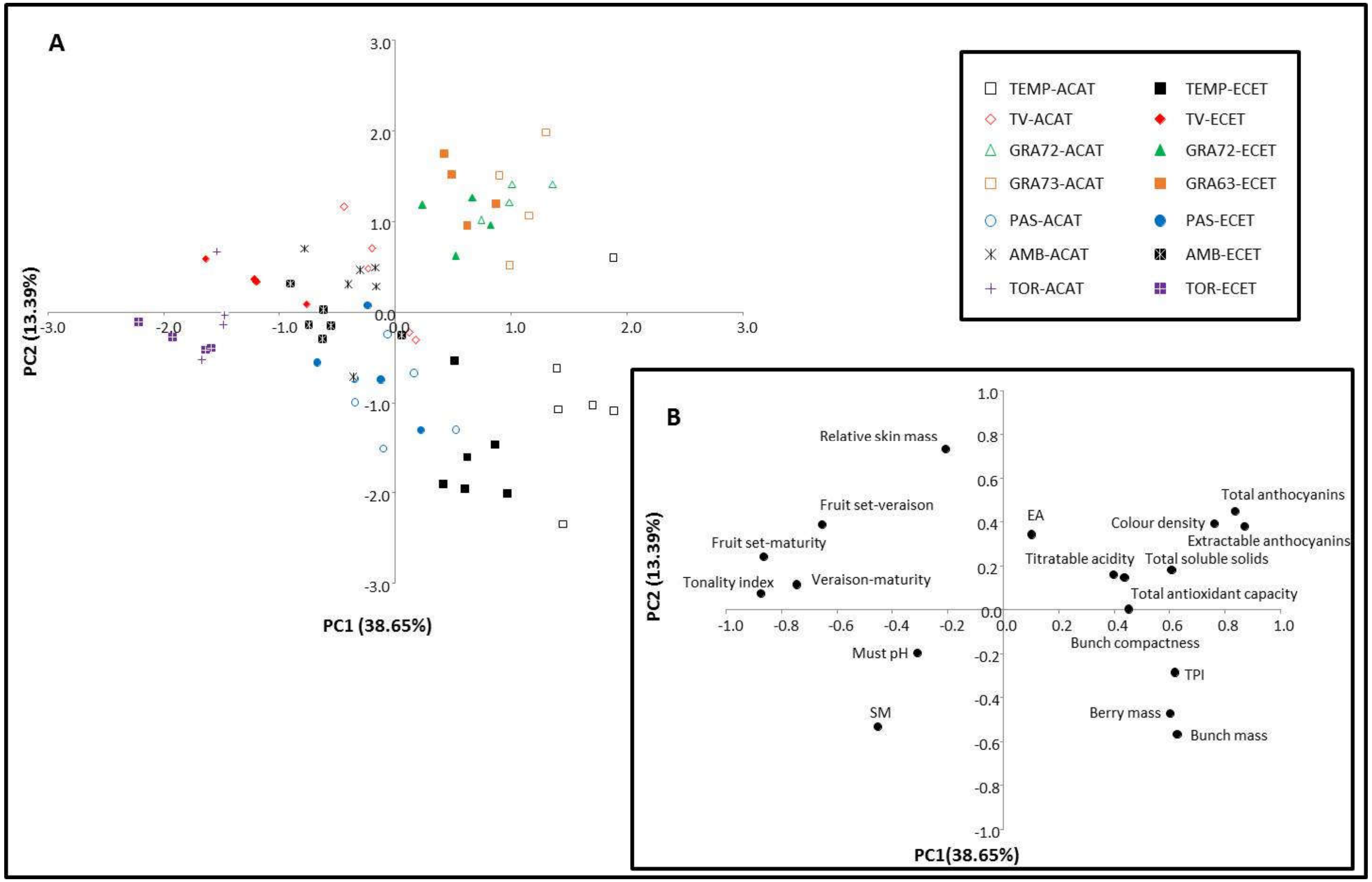

Figure S1. Principal component analysis score (A) and loading plot (B) obtained from the statistical analysis of plant and berry characteristics data of 68 studied samples from fruit-bearing cuttings of genotypes from seven local old grapevine varieties grown under two climate scenarios during berry ripening: 1) ambient CO_2 (ca. 400 µmol mol^{-1}) and ambient temperature (T) (ACAT) and 2) elevated CO_2 (ca. 700 µmol mol^{-1}) and elevated temperature (T+4°C) (ECET). The genotype labels can be found in Table 5.
